# Clinical and radiographic comparison of the effects of two types of fixed retainers on periodontium - A randomized clinical trial

**DOI:** 10.1186/s40510-014-0047-8

**Published:** 2014-08-27

**Authors:** Sepideh Torkan, Morteza Oshagh, Leila Khojastepour, Shoaleh Shahidi, Somayeh Heidari

**Affiliations:** Orthodontics Department, Shiraz University of Medical Sciences, Shiraz Orthodontic Research Center, 71345 Shiraz, Iran; Orthodontist, Private practice, 14638 Tehran, Iran; Department of Oral and Maxillofacial Radiology, Shiraz University of Medical Sciences, 71345 Shiraz, Iran; Biomaterial Research Center, Department of Oral and Maxillofacial Radiology, Shiraz University of Medical Sciences, 71345 Shiraz, Iran; Orthodontics Department, Bushehr University of Medical Sciences, 75146 Bushehr, Iran

**Keywords:** Orthodontic retainers, Clinical trial, Spiral wire, Composite resin

## Abstract

**Background:**

Most orthodontists believe that fixed retainers are necessary to maintain ideal dental relationships. However, untoward side effects might result from their long-term placement. The aim of this study was to evaluate the clinical and radiographic effect of two commonly used fixed retainers on the health of the periodontium.

**Methods:**

Thirty patients were randomly divided into two groups to receive either a fiber-reinforced composite retainer or a spiral wire retainer extended on the lingual surfaces of both maxillary and mandibular arches from canine to canine. Periapical radiographs were obtained from the patients at the time of placement of the retainers and after the 6-month period to assess the radiographic conditions of the periodontium. Clinical examination was carried out at the same two time intervals.

**Results:**

Even though there were no significant differences between the two groups of study at the beginning of the trial, there were statistically significant differences after the 6-month follow-up regarding the main outcomes of the study. Nearly all indices showed to deteriorate after 6 months in the fiber-reinforced group, while in the spiral wire group, this was not the case. As for the secondary outcomes, radiographic examination did not reveal any statistically significant differences after 6 months or between the two groups.

**Conclusions:**

It can be concluded that spiral wire retainers elicit less detrimental periodontal response in the short-term follow-up compared to fiber-reinforced composite retainers as revealed by the primary outcomes of the study.

**Trial registration:**

ClinicalTrials.gov: NCT01314729

**Electronic supplementary material:**

The online version of this article (doi:10.1186/s40510-014-0047-8) contains supplementary material, which is available to authorized users.

## Background

One of the major challenges for orthodontists is the long-term stability of orthodontic treatment. This has urged orthodontists to seek methods to ensure stable results following the completion of orthodontic treatment. Different investigators have either suggested treatment methods that can enhance the stability of treatment [1–6] or evaluated factors that can make an individual more susceptible to relapse [7–9]. However, this issue remains as a concern to both orthodontists and patients. Incisor crowding is reported to occur in follow-up stages of orthodontically treated patients [10,11]. Maintenance of mandibular incisor alignment following treatment [12] has led to the development of mandibular retainers. Such retainers may be fixed or removable [13].

Fixed mandibular retainers were first introduced in 1970s [14] and serve as a vital component of orthodontic treatment. The first generation of retainers consisted of a large diameter stainless steel round wire bonded only to the lingual side of the canines [15]. Later on, Zachrisson introduced the use of coaxial or braided wires in small diameter bonded to all mandibular anterior teeth [16,17], and most recently, fiber-reinforced composites found their way into orthodontics [18]. One of the main advantages of fiber-reinforced resin composites is their biocompatibility which is of great importance, especially in patients who are allergic to nickel component of stainless steel wires [19]. Another advantage is the esthetics, since no metal wire is present in the structure of fiber-reinforced composites [20]. In addition, it has been claimed that these retainers create a rigid splint which might contribute to a limited physiological tooth movement [21–23]. Furthermore, unless a precise fit is provided between the wire and lingual surfaces of the mandibular anterior teeth, a slight relapse in incisor irregularity might be expected [15].

Fixed retainers, in general, have been criticized for their potential to compromise the periodontal status, due to accumulation of plaque and calculus along the retainer [24,25]. The mode of functional loads that are exerted on anterior teeth also changes following splinting with fixed retainers, which might in turn compromise the health of periodontium. However, studies regarding the consequences of splinting on the health of periodontium are scarce [26,27].

Even though the effect of different orthodontic fixed retainers on periodontal status has been studied in previous investigations [13,15,24,28], to our knowledge, no studies have evaluated the fiber-reinforced composite fixed retainers or the radiographic changes in the diameter of periodontium following the placement of fixed retainers from a radiographic viewpoint. Therefore, the aims of this study were to evaluate and compare the clinical and radiographic effects of two most commonly used fixed retainers on periodontal health.

## Methods

This study was approved by the Institutional Review Board for the Health Sciences of Shiraz University of Medical Sciences and was registered under the protocol ID NCT01314729 based on the CONSORT statement [29]. This was a single-blind (to the assessor of the results), parallel-group, equality study with an allocation ratio of 1:1 conducted at a single center the Orthodontic Department of Shiraz University of Medical Sciences, Shiraz, Iran, from September 2008 to January 2010.

The sample size was calculated by a biostatistician based on the primary outcomes of the study and the significance of the results of the previous studies conducted in this field. In other words, it was based on the comparison of periodontal indices between the two groups of the study which will be explained in full details later in this section [(0.70 ± 0.62) and (1.4 ± 0.7)] with an *α* = 0.05 and power = 80% and 16 persons in each group. Estimating 20% dropouts during the trial, 40 patients were recruited for this study.

The sampling was carried out via the convenience sampling method. The patients who were in the finishing phase of treatment, willing to take part in the study and who met the inclusion and exclusion criteria of this clinical trial were selected as subjects. An informed consent was obtained from all the patients.

The inclusion criteria for the participants were as follows:

1. Healthy subjects with no history or presence of a systemic disease
2. Being in the teenage years (13 to 19 years)

The exclusion criteria were as follows:

1. Presence of any syndrome
2. Missing or extraction of any of the upper and lower anterior teeth

All the patients were treated by one orthodontist with straight wire MBT (MBT™ versatile+, 3M Unitek, Monrovia, CA, USA) 0.22 slot technique.

Within 1 to 3 weeks before the complete removal of the orthodontic appliances, the participants were referred to a dentist specialized in restorative dentistry. Each patient received either of the two fixed lingual retainers: the fiber-reinforced resin composite (fiber strand: NSI Ltd., Hornsby, Australia, composite: Filtek ™ Z250, 3M Unitek, USA) bonded on the lingual surfaces of the six upper and lower anterior teeth or a multi-stranded 0.0175-in. stainless steel wire (American Orthodontics, Sheboygan, WI, USA) bonded with a thin layer of flowable composite resin (Filtek Flow, 3M ESPE, St. Paul, MN, USA) to the six upper and lower anterior teeth. In order to randomly allocate either of the interventions to the participants of the study, block permutation design was undertaken with ten blocks including four patients in each block. The file number of all the participants eligible to take part in the study was provided for the biostatistician. Assigning each number to either of the interventions was carried out by the biostatistician. The file number and the name of the participants were also provided for the dentist who was responsible for placing the retainers. Therefore, once each patient was referred for fixed retainer insertion, the dentist allocated them to an intervention based on their numbers.

The allocation sequence was concealed from the orthodontist until after the placement of the retainer. All the participants underwent scaling and root planing, in case the presence of plaque or calculus prevented proper placement of the retainers. The decision based on which whether the patient required scaling and root planing was taken by the dentist who placed the retainers after careful examination of maxillary and mandibular anterior regions. The patients were referred for the placement of retainer since the restorative dentist was more experienced in placing the retainers than the orthodontist. For each patient, a wire was formed chairside by the orthodontist and was sent to the dentist to minimize any mistakes in forming the wire due to the lack of experience by the dentist. If the patient was allocated to the spiral wire group, the wire would be used by the dentist; otherwise, fiber-reinforced composite would be placed.

Once the retainers were placed, the participants were instructed by the same dentist who placed the retainers to undertake meticulous oral hygiene using both superfloss and interdental brushes along with tooth brushing and to avoid visiting another dentist for any purpose other than caries check-up until the recall appointment.

Upon removal of the orthodontic appliances and 6 months after the insertion of the retainers, the participants were checked for their periodontal health along the retainer based on different clinical indices. The assessment and scoring of the indices were carried out by a calibrated dentist.

To assess the periodontal health from a radiographic viewpoint, three digital periapical radiographs (two from the upper anterior teeth and one containing the lower anterior teeth) were obtained from each individual upon completion of the treatment and after a 6-month interval. The radiographs were obtained by the same radiologist through the parallel cone technique with a Rinn XCP holder (Dentsply, York, PA, USA).

The following clinical variables were measured on each individual as the main outcome measures:

1. *Plaque index* (PI), as described by Loe [30], was evaluated using a disclosing agent on the lingual surfaces of all maxillary and mandibular anterior teeth.

Plaque accumulation was categorized using the following scale:

0 - Absence of plaque deposits
1 - Plaque disclosed after running the periodontal probe along the gingival margin.
2 - Visible plaque
3 - Abundant plaque

The results were averaged for the six maxillary and mandibular teeth, and a mean value was recorded for the maxilla and mandible in each participant.

2. *Calculus index* (CI) [31,32] using the following scale:
  0 - Absence of calculus
  1 - Presence of calculus covering up to one third of the tooth surface
  2 - Presence of calculus covering up to two third of the tooth surface and/or the presence of separate flecks of subgingival calculus
  3 - Presence of calculus covering more than two third of the tooth surface and/or presence of a continuous band of subgingival calculus

An average was calculated for mandibular and maxillary teeth and the mean value was recorded.

3. *Gingival index* (GI) suggested by Loe [30] includes the following:
  0 - Absence of inflammation
  1 - Mild inflammation, slight change in color, little change in texture, no bleeding on probing
  2 - Moderate inflammation, moderate glazing, redness, edema and hypertrophy, bleeding on probing
  3 - Severe inflammation, marked redness, hypertrophy, tendency for spontaneous bleeding, ulceration

GI was only assessed on the lingual surfaces so that the presence of any labial inflammation does not affect the results.

4. *Bleeding on probing* (BOP) was measured 15 s after the insertion of a Florida probe [33] into the gingival crevice [34]. A standardized pressure of 25 g was used [35,36] to eliminate operator bias. The following scores were assigned to each tooth in the anterior segment of the upper and lower arch:
  0 - No bleeding
  1 - Bleeding on probing

Since all the indices were to be evaluated on the lingual surfaces of the teeth, the presence of bleeding following the removal of orthodontic appliances on the labial surfaces of the teeth did not play a role in the scoring results.

The secondary outcomes of the study were evaluated by assessing the PDL width which is defined as the maximum width of the PDL space of the teeth measured on parallel periapical radiographs. Radiographs were assessed by two radiologists with more than 10 years of clinical experience. The radiologists were blinded to the study and therefore not aware whether the radiographs were taken at the beginning of the trial or after 6 months. They were asked to score the radiographs relatively, that is, for each patient, the periapical radiograph of the 0 and 6 months of each segment were randomly numbered and shown to each radiologist; they were then asked to mark the number they felt had the largest PDL width. After 1 month, they were asked to repeat the process. Based on the reports of the two radiologists, if the PDL width had remained unchanged or had increased over the 6-month period, a negative value was assigned to that segment; however, had the PDL width decreased in diameter, a positive value was assigned to the periapical radiograph of that segment. This classification was based upon this premise that the teeth must have regained their normal PDL width by the final appointment (6 months after completion of orthodontic treatment) [37,38]; therefore, either a widening or a steady state in the width of PDL could indicate an undesirable situation. It was speculated that if a positive value was documented, it could mean that reorganization is in progress or has taken place, while a lack of change or widening recorded by a negative value could indicate that splinting the teeth has an inhibitory effect on the reorganization of the periodontal fibers. It is a speculation that is yet to be verified in future studies.

### Statistical analysis

In order to evaluate the primary outcome measures, Wilcoxon test was used to evaluate the changes in each group between the 0- and 6-month period. To assess the difference between the two groups, Mann-Whitney test was used. To assess intra- and inter-examiner reliability in scoring periapical radiographs, Kappa coefficient was used. As for assessing the secondary outcome measures, Fisher's exact test was used.

## Results

Forty patients were randomly selected for this clinical trial and were divided into two groups. Ten patients were withdrawn from the study either because they missed their final appointment (*n* = 3 in the spiral wire group and *n* = 2 in the fiber-reinforced group) or because a breakage was diagnosed along the retainer. For stability purposes, these patients had to be immediately referred for the placement of the new retainer. The need for scaling and root planing was assessed and was carried out if required in these patients. Therefore, they had to be withdrawn from further evaluation since the subjects were not to receive any professional cleaning during the 6-month follow-up period. The total sample size therefore was 30 (15 in each group) (see Additional files 1 and 2).

Table 1 outlines the demographic data of the participants in the study. For both study groups, the median and interquartile ranges for all the measured indices (primary outcome measures) immediately and 6 months after the insertion of the retainers are presented in Table 2. In Tables 3 and 4, the differences between the 0 and 6 months between the two study groups for the clinical indices are outlined in maxillary and mandibular arches, respectively.

**Table 1:** **Demographic data and clinical characteristics of the participants of the study**

		**Study groups**			
		**SW (** ***n*** **= 15)**		**FRC (** ***n*** **= 15)**	
		**Mean or %**	**SD**	**Mean or %**	**SD**
Gender (%)	Male	26.6		40	
	Female	73.4		60	
Age (years)		15.7	2.1	16.2	1.9
Treatment	Extraction	46.6		40	
	Non-extraction	53.4		60	

SW, spiral wire group; FRC, fiber-reinforced composite group.

**Table 2:** **Median and interquartile range values for measured clinical indices in both fiber-reinforced and spiral wire group**

			**Baseline**		**6 months**		***P*** **value**
			**Median**	**Interquartile range**	**Median**	**Interquartile range**	
Plaque index	SW	Maxilla	0.33	1	0.66	1	0.16
		Mandible	0.33	1	0.91	1	0.077
	FRC	Maxilla	0.0	1	1.66	1	0.003*
		Mandible	0.91	1	2.0	0	0.001*
Gingival index	SW	Maxilla	0.0	0	0.83	1	0.01*
		Mandible	0.16	1	0.41	1	0.56
	FRC	Maxilla	0.5	1	1.0	1	0.032*
		Mandible	0.33	2	1.0	1	0.021*
Calculus index	SW	Maxilla	0.0	0	0.0	1	1
		Mandible	0.0	0	0.0	0	0.042*
	FRC	Maxilla	0.0	0	0.0	0	0.15
		Mandible	0.0	0	0.33	0	0.018*
Bleeding on probing	SW	Maxilla	0.0	0	0.5	1	0.045*
		Mandible	0.0	1	0.33	1	0.16
	FRC	Maxilla	0.16	0	0.5	1	0.044*
		Mandible	0.0	1	0.66	1	0.025*

SW, spiral wire group; FRC, fiber-reinforced composite group.

*, statistically significant.

**Table 3:** **Comparison of clinical indices between two study groups at baseline and after 6 months in maxillary arch**

**Variable measured**		**FRC baseline**	**FRC 6 months**
Plaque index	SW 6 months	-	0.015*
	SW baseline	0.46	-
Gingival index	SW 6 months	-	0.044*
	SW baseline	0.51	-
Calculus index	SW 6 months	-	0.53
	SW baseline	1	-
Bleeding on probing	SW 6 months	-	0.35
	SW baseline	0.54	-

SW, spiral wire group; FRC, fiber-reinforced composite group.

*, statistically significant.

**Table 4:** **Comparison of clinical indices between two study groups at baseline and after 6 months in mandibular arch**

**Variable measured**		**FRC baseline**	**FRC 6 months**
Plaque index	SW 6 months	-	0.001*
	SW baseline	0.62	-
Gingival index	SW 6 months	-	0.000*
	SW baseline	0.53	-
Calculus index	SW 6 months	-	0.35
	SW baseline	074	-
Bleeding on probing	SW 6 months	-	0.07
	SW baseline	0.86	-

SW, spiral wire group; FRC, fiber-reinforced composite group.

*, statistically significant.

The kappa coefficient for the intra-examiner reliability in assessing the PDL width was 0.81 which is excellent [39]. Also, the kappa coefficient for inter-examiner reliability was 0.86 which reports excellent agreement (*P* < 0.001).

None of the indices revealed any significant differences between the two groups of study at the baseline, neither in maxillary nor in the mandibular arch, which implements similar periodontal status in all the individuals at the beginning of the trial. However, after 6 months, there were significant differences in gingival index and plaque index between the spiral wire and fiber-reinforced composite groups in both arches. In addition, a significant difference was found to exist in the formation of calculus in the mandibular arch after 6 months of follow-up in both study groups. All the scores showed to deteriorate after 6 months in both study groups. However, the scores recorded in the fiber-reinforced group reported worse periodontal status compared to the spiral wire group. The calculus index and bleeding on probing showed no statistically significant difference after 6 months between the two groups.

Fischer exact test compared the results of periapical radiographs but failed to reveal any differences between the two groups. This might be attributed to the number of the samples in the study. It was shown that 64% of the maxillary radiographs in the spiral wire group reported a positive value, while in 60% of the maxillary periapical radiographs in the fiber-reinforced group, the PDL had either widened or remained unchanged in its diameter. The differences, though, were not statistically significant. For mandible, these results were 50% and 60%, respectively.

## Discussion

In this prospective clinical trial, the effect of two common types of fixed retainers on the health of the periodontium was evaluated. All the study samples were selected within the same age group, as different periodontal issues such as gingival recession tends to be affected with age [15].

Even though it has been concluded that following orthodontic treatment, inflammatory responses do follow [40], all the patients in the present study were treated with multibonded appliances which do not seem to affect the score results in the lingual areas examined right after the removal of orthodontic appliances [13].

The results of our study showed a significant difference in the accumulation of plaque between the two study groups after 6 months, with worse scores recorded in the fiber-reinforced group. This might be attributed to the fact that fiber-reinforced composites occupy a wider span on the lingual surface of the teeth compared to spiral wire retainers. Even though there was no significant difference in the calculus index between the two groups after the 6-month follow-up, there was a significant increase in the accumulation of calculus in the mandibular arch after the 6-month follow-up in both study groups. This finding is not in agreement with the results of Artun et al. who concluded there were no differences in plaque or calculus accumulation between retainers made of spiral wire and those made of plain wire [24,28]. In addition, Booth et al. reported 0.25-in. steel wire placed from canine to canine as retainer did not affect the quality of oral hygiene maintenance [13]. Heier et al. compared bonded spiral wires with removable retainers and did not report a significant difference in gingival inflammation in the two groups. However, more calculus was found to exist around fixed retainer after 6 months [41]. It has to be stated that one would expect the same results in the lower anterior region of untreated control subjects compared to upper anterior region, due to limited access for maintenance of oral hygiene and the proximity of the lower incisal segment to the opening of the submandibular and sublingual salivary glands.

When each retainer was evaluated separately, there was a significant difference between the baseline and 6-month follow-up in the spiral wire group for both the gingival index and bleeding on probing in the maxillary arch and calculus index in the mandibular arch.

In the fiber-reinforced composite group, all the measured indices increased in both arches after the 6-month follow-up. The calculus index, however, showed no statistically significant differences in the maxillary arch. This can be attributed to the wider clinical crowns of maxillary incisors which provide better access for oral health maintenance compared to mandibular incisors.

It has been suggested that placing fixed orthodontic retainers on the maxillary arch poses more risks on the periodontal health, since the retainer has to be placed gingival enough to avoid premature occlusal contacts [15]; however, in the present study, there were no significant differences between the scores recorded for the maxillary arch between the baseline and after the 6-month follow-up.

It has been claimed that fiber-reinforced composite retainers provide a smoother outer surface, while there seems to be more retention areas along the spiral wire retainers which can contribute to the formation of more calculus along the retainer wire [15,24]. In our study, however, in both groups, there was a significant increase in the accumulation of the calculus in the mandibular arch with the mean scores being worse in the fiber-reinforced group.

Fiber-reinforced composites were recently introduced into dentistry and soon found their way into orthodontics as fixed retainers. Fibers were added to composites on the premise of providing more rigidity to the composites [42]. This advantage can also serve as a disadvantage in orthodontics where it might limit physiologic tooth movement and therefore compromise the health of periodontium. It is claimed that multistranded wires allow physiologic tooth movement to occur which is also required for reorganization of PDL fibers [21,43]. During the first 3 to 4 months after the completion of orthodontic treatment, full-time retention is mandatory to prevent relapse while the PDL fiber reorganization occurs [43]. It has also been well documented that PDL widens remarkably during the orthodontic treatment [37]; therefore, it seems logical that if a certain type of retainer is to provide physiologic tooth movement, after 6 months of follow-up, PDL should have regained its normal width. Based on this hypothesis, a positive value was assigned to the periapical radiographs in which the width of the PDL was decreased after the 6-month follow-up, indicating the normal reorganization process of PDL fibers; otherwise, a negative value was assigned to the periapical radiograph. For this purpose, periapical radiographs were obtained from each individual, both at the baseline and after the 6-month follow-up. Even though there has been some criticism over the reliability of assessing the width of periodontal ligament via the periapical radiographs [15], periapical radiographs have long been considered as the standard screening method. Based on this agreement, it was observed that in the maxillary arch, for 60% of the individuals in the fiber-reinforced group, a negative value was registered. This reported percentage for the spiral wire group was 35%. The difference between the two groups was not statistically significant. This can be attributed to the number of the individuals examined in this study. These results reveal that in more than half of the patients in the fiber-reinforced group, PDL had not regained its normal width following the completion of orthodontic treatment. The recorded percentage in the fiber-reinforced group was almost the same for mandibular arch, but in the spiral wire group, it was increased to 50%. It can be presumed that the presence of fixed retainer does not permit full reorganization of PDL fibers after 6 months of follow-up. To our knowledge, none of the previous studies have evaluated the changes in the PDL width following insertion of fixed orthodontic retainers. However, Pandis et al. reported no change in the bone level following the placement of lingual fixed retainers, even though probing depth was increased [15]. However, in their study, all the retainers were fabricated by 0.195-in. braided wire. Furthermore, the changes in the PDL are reported to occur at an earlier stage of periodontal disease than the changes in alveolar crest [44] and thus need to be taken into account more readily.

It can be suggested that in future studies, a control group with removable retainers be added to the study groups. The number of patients included in each study group can be further increased. If cone beam computed tomography finds its way to orthodontics as a routine pre-treatment diagnostic record, it can be further used as a precise tool for assessing the changes in the periodontal ligament. However, due to the increased radiation risk and excessive cost, its use cannot be justified for the sole purpose of assessing the periodontal width. The follow-up duration can be increased up to 2 years as fixed retainers are usually left in place for periods longer than 6 months.

## Conclusions

The results of this study showed that when the fiber-reinforced composite retainers and spiral wire retainers were compared, less detrimental side effects were observed after 6 months in the spiral wire retainers. From the radiographic viewpoint, the effect of the retainers on the health of the periodontium was statistically insignificant and inconclusive.

## Additional files

Additional file 1:
**CONSORT 2010 Flow Diagram.** This file summarizes the general data regarding the enrollment of the subjects, number of subjects allocated to each treatment and the final number of subjects analyzed. Additional file 2:
**CONSORT 2010 checklist of information to include when reporting a randomised trial*.** This file outlines the different sections of a randomized clinical trial and their designated page in the article.
