# Supplementary figures and images for: Clinical and radiographic comparison of the effects of two types of fixed retainers on periodontium - A randomized clinical trial
Source: Prog Orthod. 2014 Aug 27;15(1):47. doi: 10.1186/s40510-014-0047-8 (PMC4145221; doi:10.1186/s40510-014-0047-8)

## CONSORT 2010 Flow Diagram

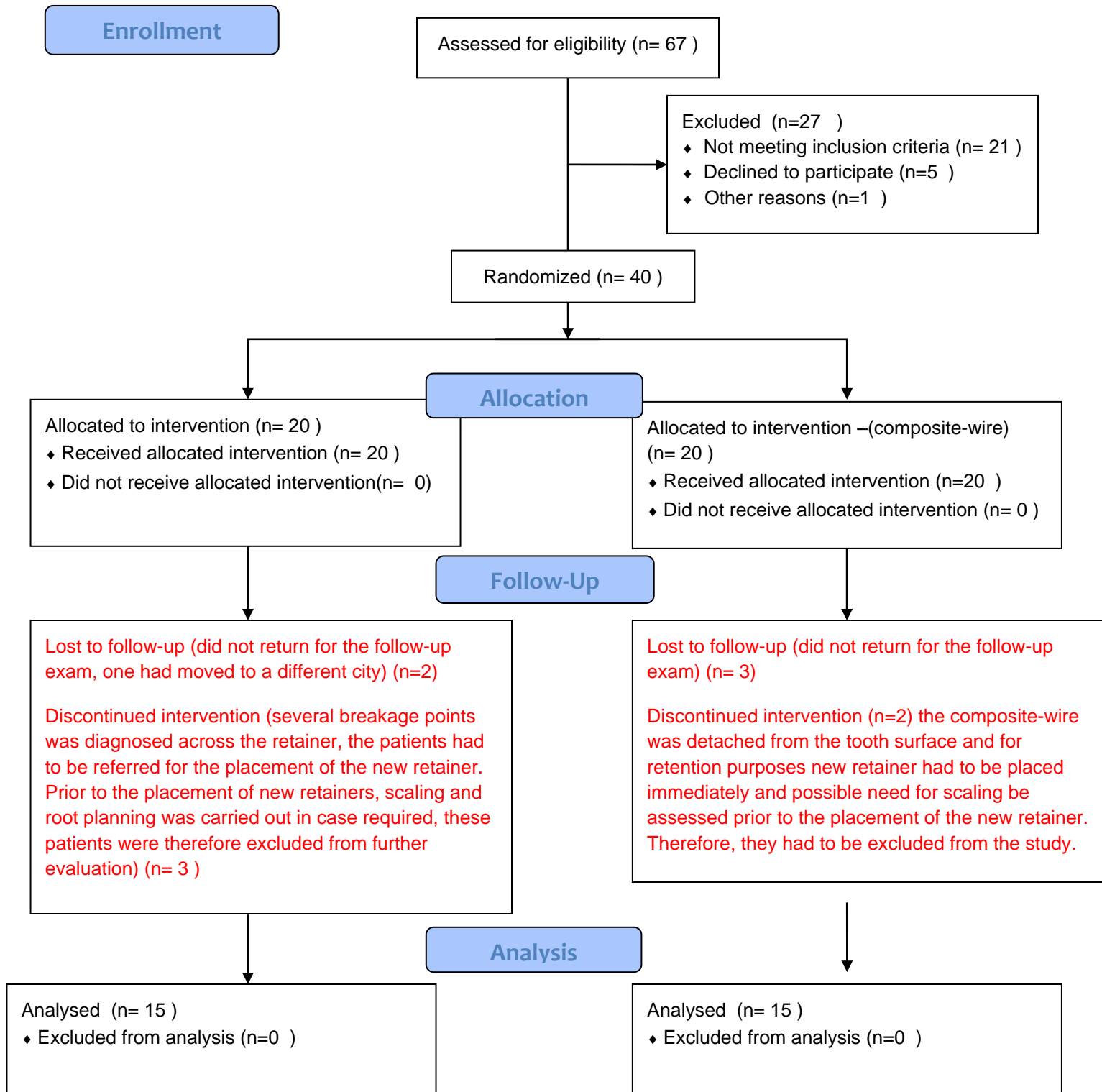

Supplement: Additional file 1: — CONSORT 2010 Flow Diagram. This file summarizes the general data regarding the enrollment of the subjects, number of subjects allocated to each treatment and the final number of subjects analyzed. [file 40510_2014_47_MOESM1_ESM.pdf]
